# Timing for cranioplasty to improve neurological outcome: A systematic review

**DOI:** 10.1002/brb3.1106

**Published:** 2018-10-02

**Authors:** Maria C. De Cola, Francesco Corallo, Deborah Pria, Viviana Lo Buono, Rocco S. Calabrò

**Affiliations:** ^1^ IRCCS Centro Neurolesi “Bonino Pulejo” Messina Italy

**Keywords:** cognitive outcomes, cranioplasty, motor recovery, neurorehabilitation

## Abstract

**Introduction:**

Cranioplasty is a surgical technique applied for the reconstruction of the skullcap removed during decompressive craniectomy (DC). Cranioplasty improves rehabilitation from a motor and cognitive perspective. However, it may increase the possibility of postoperative complications, such as seizures and infections. Timing of cranioplasty is therefore crucial even though literature is controversial. In this study, we compared motor and cognitive effects of early cranioplasty after DC and assess the optimal timing to perform it.

**Methods:**

A literature research was conducted in PubMed, Web of Science, and Cochrane Library databases. We selected studies including at least one of the following test: Mini‐Mental State Examination, Rey Auditory Verbal Learning Test immediate and 30‐min delayed recall, Digit Span Test, Glasgow Coma Scale, Glasgow Outcome Scale, Coma Recovery Scale‐Revised, Level of Cognitive Functioning Scale, Functional Independence Measure, and Barthel Index.

**Results:**

Six articles and two systematic reviews were included in the present study. Analysis of changes in pre‐ and postcranioplasty scores showed that an early procedure (within 90 days from decompressive craniectomy) is more effective in improving motor functions (standardized mean difference [SMD] = 0.51 [0.05; 0.97], *p*‐value = 0.03), whereas an early procedure did not significantly improve neither MMSE score (SMD = 0.06 [−0.49; 0.61], *p*‐value = 0.83) nor memory functions (SMD = −0.63 [−0.97; −0.28], *p*‐value < 0.001). No statistical significance emerged when we compared studies according to the timing from DC.

**Conclusions:**

It is believed that cranioplasty performed from 3 to 6 months after DC may significantly improve both motor and cognitive recovery.

## 1 INTRODUCTION

Decompressive craniectomy (DC), consisting in the partial removal of the skullcap, is widely used in the management of neurological emergencies as it allows a decrease in brain swelling and intractable intracranial hypertension (Hofmeijer et al., 2009). DC is performed for a variety of reasons, but the most common are tumor removal and the reduction in increased intracranial pressure due to malignant ischemic or hemorrhagic stroke (Hofmeijer et al., 2009; Vahedi et al., 2007). Cranioplasty (CP) is a neurosurgical procedure aimed to repair the skull defect following craniectomy.

The search for materials and strategies to provide more comfortable and reliable surgical procedures is a challenging topic, both in clinical and in economical terms. However, none of the currently available materials meets the criteria required for an ideal implant (Zanotti et al., 2016).

Besides a purely aesthetic reason, CP helps the individual's rehabilitation from different points of view. The possible advantages of CP have been discussed extensively in literature, as increased cerebral blood flow (Coelho et al., 2014; Erdogan et al., 2003; Maekawa, Awaya, & Teramoto, 1999), change in cerebrospinal fluid hydrodynamics (Juul, Morris, Marshall, & Marshall, 2000; Mah & Kass, 2016; Winkler, Stummer, Linke, Krishnan, & Tatsch, 2000), and reduction in epileptic seizures (Nalbach, Ropper, Dunn, & Gormley, 2012). Recently, promising results following this procedure in both motor and cognitive outcomes have been reported. Thus, this link between the repair of the cranial defect and the changes in cerebrovascular and cerebrospinal fluid hydrodynamics seems to have positive effects on neurological functions (Bijlenga, Zumofen, Yilmaz, & Creisson, 2007).

If on one hand CP may lead to notable improvements (Sancisi et al., 2009; Stiver, Wintermark, & Manley, 2008), on the other hand it may increase the possibility of infections, the risk of hydrocephalus (especially when performed later), and the possibility of developing the “trephined” syndrome (Stiver, Wintermark, & Manley, 2008), especially when operation time exceeding 90 min (Cho & Kang, 2017). Indeed, although the mortality rate after cranioplasty is rather low, research suggests that 1 out of 3 people has overall complications (Zanaty et al., 2015), especially seizures and infection (Honeybul & Ho, 2016). Timing of cranioplasty is therefore crucial even though the literature is divided. According to several studies, it should be performed from 3 to 12 months following DC, based on the presence of infections or postoperative complications. Indeed, in order to prevent the development of devitalized autograft or allograft infections it is recommended to wait from 3 to 6 months before reconstructive surgery, even one year if there is an infected area (Aydin, Kucukyuruk, Abuzayed, Aydin, & Sanus, 2011). On the contrary, an early intervention (i.e., within 3 months) seems to reduce neurological complications, especially in patients with severe acquired brain injury, since a lesion in the postacute period might be negative for motor and cognitive recovery (Huang, Lee, Yang, & Liao, 2013). Although the timing to perform cranioplasty largely depends on personal clinical experience rather than evidence‐based data, it could be useful to estimate a suitable threshold to perform cranioplasty.

In this article, we want to review current literature on motor and cognitive effects of an early cranioplasty after decompressive craniectomy, also focusing on the optimal timing to perform it.

## 2 METHODS

### 2.1 Data sources and keywords

A systematic review and meta‐analysis in line with the Preferred Reporting Items for Systematic Reviews and Meta‐Analyses (PRISMA) guidelines were performed.

Articles published up to July 2017 were searched on the PubMed, Web of Science, and Cochrane Library databases, without language restrictions. A follow‐up search was done in January 2018. Databases were queried using key words, and their combinations as follows: “Recovery AND Cranioplasty”; “Rehabilitation AND Cranioplasty”; “Timing AND Cognitive AND Cranioplasty”; “Timing AND Motor AND Cranioplasty”; “Early AND Cognitive AND Cranioplasty”; “Early AND Motor AND Cranioplasty”; “Cognitive recovery AND Cranioplasty”; “Motor recovery AND Cranioplasty.”

### 2.2 Study selection and search strategy

All studies reporting motor and/or cognitive recovery after cranioplasty for the patients with cranial defects after DC were included. Systematic reviews that investigated the effects of cranioplasty timing on motor and cognitive recovery in patients underwent cranioplasty were also included. Reports of less than ten subjects, comments, letters, editorial articles, and studies included mainly patients <18 years old were excluded.

At first, search results were summarized and duplicate citations were deleted, together with non‐English articles. Then, titles were screened for relevance to motor and cognitive recovery after cranioplasty. Next, abstracts of the remaining articles were read and those not meeting the eligibility criteria were excluded. The full text of all potential articles was evaluated in depth. In case of uncertainty, or when the abstract was not available, the entire article was read. Two reviewers performed independently the selection of the articles included in this systemic review. The Cohen's kappa score for inter‐rater agreement in study selection was computed (Sands & Murphy, 1996). Discrepancy was resolved through discussion.

### 2.3 Data extraction and outcomes

Data from the studies were collected in an electronic sheet including age, gender, pathology, craniectomy to cranioplasty time interval, surgical site, and pre‐ and postcranioplasty assessment. Concerning the latter, given that our primary outcome was to compare effects of early and late cranioplasty on the cognitive and motor recovery, we selected studies including as assessment tools Mini‐Mental State Examination (MMSE; Folstein, Folstein, & McHugh, 1975), Rey Auditory Verbal Learning Test immediate (RAVLT) and 30‐min delayed recall (RAVLT‐DR; McMinn, Wiens, & Crossen, 1988), and Digit Span Test (DST; Schroeder, Twumasi‐Ankrah, Baade, & Marshall, 2012). We considered patients with disorders of consciousness separately, including studies reporting data from the Glasgow Coma Scale (GCS; Doyle, 1989), the Glasgow Outcome Scale (GOS; Wilson, Pettigrew, & Teasdale, 1998), the Coma Recovery Scale‐Revised (CRS‐R; Giacino, Kalmar, & Whyte, 2004), and the Level of Cognitive Functioning Scale (LCF; Sander, 2012). Concerning the motor recovery, we selected studies including Functional Independence Measure (FIM; Keith, 1987) or Barthel Index (BI; Collin, Wade, Davies, & Horne, 1988).

In absence of at least one of the aforementioned assessments, administered both at baseline and at follow‐up, we excluded the article from the meta‐analysis for inadequate study design.

### 2.4 Data analysis

The meta‐analysis was performed using the metafor package of R (version 3.4.0; the R Foundation for Statistical Computing, Vienna, Austria), setting at *α* = 0.05 the statistical significance. Statistical averages and relative percentages of all patient characteristics were combined, when and if appropriated. The main analysis concerned the effects of early versus late cranioplasty on motor and cognitive recovery, assessed by comparing the changes in pre‐ and postcranioplasty scores. For studies reporting multiple test assessment, only the primary outcome was included in the analysis. For studies reported multiple evaluation times before CP, we considered as pre‐CP evaluation the one closest to the date of the procedure.

We also performed a subgroup analysis by subdividing the studies according to the time interval from DC to CP: within 3 months and within 6 months. Where the article included both the early and the late cranioplasty groups, we considered the patient's subdivisions of the original study. Otherwise, we subdivided the patients into two groups choosing a threshold according to the median time interval between DC and CP.

Since many studies used different outcome scales, as well as had different sample dimensions, the treatment effect of an intervention was estimated by pooling the standardized mean difference (SMD) with 95% confidence interval (CI). Heterogeneity was quantified by the estimated between‐study variance *τ* ^2^, *I* ^2^. When the level of heterogeneity was higher than 75%, we considered the results obtained by the application of the random‐effects model. Risk of bias, at outcome level, was graphically investigated by funnel plot.

## 3 RESULTS

### 3.1 Study selection

**Figure 1:**
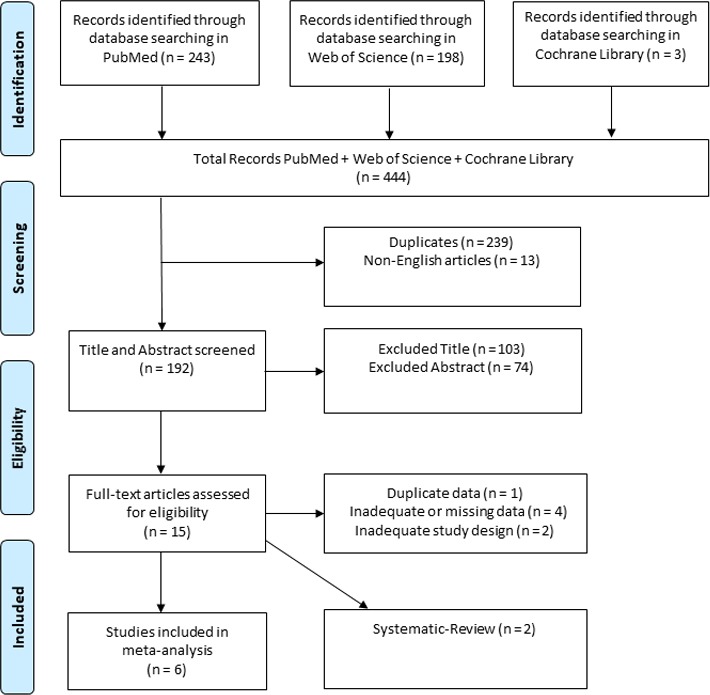
PRISMA flow diagram describing the study selection process

Figure 1 shows our study selection process. A total of 444 records were identified: 243 articles from PubMed database, 198 articles from Web of Science database, and three articles from the Cochrane Library database. After removing 239 duplicates and 13 non‐English articles, 192 articles were identified. Later, 103 records were excluded by reading titles and 74 articles by reading abstract, including all case‐report and case–control studies for unsustainability of results. The case reports and/or case series studies excluded were summarized for the purpose of this systematic review in Table 1.

**Table 1:** Overview of case report excluded in this review

Study	Age (years)	Gender	Etiology	Surgical site	Time DC‐Cranioplasty	Complications after		Motor outcome	Cognitive outcome	Time between assessment
						Decompressive Craniectomy	Cranioplasty			
Alibhai, Balasundaram, Bridle, and Holmes (2013)^a^	79	Male	Tumor	Unilateral (R)	–	–	Seizure	–	Improved	8 weeks
Corallo, Calabro, Leo, and Bramanti (2015)	55	Male	Vascular	Unilateral (R)	8 months	VS, Partial seizure	–	Improved	Improved	6 months
Ratnasingam, Lovick, Weber, Buonocore, and Williams (2015)	21	Male	TBI	Bifrontal	6 months	–	Seizure, Bells' palsy	–	Improved	26 months
Jeyaraj (2015)	52	Male	TBI	Unilateral (L)	3 months	Hemiparesis DX, Hydrocephalus, Syndrome of the trephined		Improved	Improved	11 days
Nguyen, Doan, Gelsomino, Shabani, and Mueller (2016)	37	Male	Vascular	Unilateral (L)	3 months	–	–	Improved	Improved	–
Corallo, Marra, Bramanti, and Calabrò (2014)	30	Male	Vascular	Unilateral (R)	50 days	Hemiparesis SX with dysesthesia, depression, anhedonia, irritability, sleep alterations	–	Improved	Improved	3 months
Castaño‐Leon et al. (2017)	36	Male	TBI	Bilateral	7 months	Hydrocephalus, cephalea, dizziness, vomiting, diplopia	None	Improved	Improved	–
Segal, Oppenheim, and Murovic (1994)	35	Male	TBI	Bilateral	6 months	Blind, left leg paretic, right leg plegic, left hand plegic.	None	Improved	–	7 days

a We reported 1 case out of 2 patients. TBI: Traumatic brain injury.

The 15 remaining studies were full‐text‐screened. After reading them, one article was excluded because reported duplicate patients (Honeybul, Janzen, Kruger, & Ho, 2013), who were included in a more recent and larger simple size study (Honeybul, Janzen, Kruger, & Ho, 2016), whereas two articles were excluded for inadequate study design (Huang et al., 2013; Liang et al., 2007). Although we contacted eight authors for further information regarding missing data (Bender et al., 2013; Di Stefano et al., 2016; Honeybul et al., 2016; Jasey, Ward, Lequerica, & Chiaravalloti, 2018; Shahid, Mohanty, Singla, Mittal, & Gupta, 2018; Songara, Gupta, Jain, Rege, & Masand, 2016; Stelling, Graham, & Mitchell, 2011; Su et al., 2017), only four of them were able to provide original individual data useful for our meta‐analysis (Di Stefano et al., 2016; Honeybul et al., 2016; Jasey et al., 2018; Songara et al., 2016). At the end of selection, six articles and two systematic reviews (Malcolm et al., 2018; Xu et al., 2015) have been included in the present study.

The kappa score for inter‐rater agreement in study selection was 0.88 indicating an “almost perfect agreement,” (Landis & Koch, 1977) with a percentage of agreement between the two reviewers of 99.3%.

### 3.2 Study characteristics

In Table 2 are reported the six studies included in quantitative analysis, with a total of 162 patients (70.99% males and 29.01% females), whereas in Table 3 are reported the seven excluded studies.

**Table 2:** Characteristics of included studies

Study	Type	Etiology	Surgical site	Early cutoff	Early CP				Late CP				Motor outcome	Cognitive outcome	Follow‐up
					*N*	Age (years)	Male	DC‐CP time interval (d)	*N*	Age (years)	Male	DC‐CP time interval (d)			
Honeybul et al. (2016)	Retrospective	TBI (72%) Vascular (20%) Tumor (6%)	Bifrontal (28.0%) Unilateral R (34.0%) Unilateral L (38.0%)	3 months	20	45.5 ± 16.6	16	64.0 ± 15.2	30	37.2 ± 16.0	22	157.0 ± 125.5	FIM	DST	<3 days
Stefano et al. (2016)	Prospective	TBI (72.5%) Vascular (27.5%)	Bilateral (24.2%) Unilateral R (31.0%) Unilateral L (37.9%) NA (6.9%)	6 months	15	39.1 ± 15.6	12	127.9 ± 31.9	14	41.0 ± 10.9	10	399.9 ± 85.9	–	RAVLT, RAVLI‐D, DST	1 month, 6 months^a^
Corallo et al. (2017)	Prospective	TBI (40%) Vascular (60%)	Bilateral (20.0%) Unilateral R (53.3%) Unilateral L (26.7%)	6 months	15	51.5 ± 15.5	6	4.5 ± 3.0	15	51.1 ± 12.8	11	7.9 ± 3.5	–	MMSE, GCS, DST, RAVLI RAVL‐DR, CRS‐R, LCF	1 month, 1 year^a^
Songara et al. (2016)	Prospective	TBI (100%)	Bilateral (6.2%) Unilateral R (31.3%) Unilateral L (62.5%)	3 months	6	34.5 ± 14.6	4	63.7 ± 16.4	10	38.7 ± 12.0	10	195.8 ± 104.9	–	MMSE, GCS, GOS	1 week^a^, 4 weeks
Kim et al. (2017)	Retrospective	TBI (50%) Vascular (50%)	NA	3 months	12	58.7 ± 15.5	7	74.0 ± 14.5	12	51.4 ± 13.1	8	219.0 ± 131.3	FIM, K‐MBI^a^	K‐MMSE	<4 weeks
Jasey et al. (2018)	Retrospective	TBI (69%) Vascular (31%)	Bilateral (7.7%) Unilateral R (61.5%) Unilateral L (30.8%)	3 months	5	40.8 ± 17.8	3	75.4 ± 19.4	8	45.5 ± 19.2	6	135.5 ± 33.7	FIM	–	NA

Notes

MMSE: Mini‐Mental State Examination; K‐MMSE: Korean Mini‐Mental State Examination; RAVLT: Rey Auditory Verbal Learning Test immediate; RAVLT‐DR: and 30‐min delayed recall; DST: Digit Span Test; GCS: Glasgow Coma Scale; GOS: Glasgow Outcome Scale; CRS‐R: Coma Recovery Scale‐Revised; LCF: Level of Cognitive Functioning Scale; FIM: Functional Independence Measure; K‐BI: Korean Barthel Index; TBI: traumatic brain injury; NA: Not Available.

a Not considered in meta‐analysis.

**Table 3:** Characteristics of full‐text excluded studies

Study	Type	Etiology	Surgical site	Patients	Age, years	Male	Time interval from DC to CP	Motor outcomes	Cognitive outcomes	Follow‐up
Honeybul et al. (2013)	Retrospective	TBI (76.0%) Vascular (20.0%) Tumor (4%)	Bifrontal (40%) Unilateral R (32%) Unilateral L (28%)	25	40 (25–59)	19 (76%)	100 ± 128 days	FIM	DST	<3 days
Bender et al. (2013)	Retrospective	TBI (46.2%) Vascular (51.8%)	Bifrontal (5%) Unilateral R (50%) Unilateral L (45%)	147	48.3 ± 16.8	95 (64.6%)	86.4 ± 129.7 days	BI, FIM	CRS‐R	161.7 ± 68.3 days
Stelling et al. (2011)	Retrospective	TBI (65.0%)	NA	23	Mean 37 16–64 ranged	16 (69.6%)	12 days to 35 months	–	GCS, GOS	<15 months
Shahid et al. (2018)	Prospective	TBI (100.0%)	Bifrontal (2.9%) Unilateral R (50%) Unilateral L (47.1%)	34	31.53 ± 10.08	30 (88.2%)	Mean 5 months 3–29 ranged	–	GCS (pre), GOS, RAVLT, RAVLT‐DR, DST	3 months
Huang et al. (2013)	Retrospective	TBI (100.0%)	NA	105	41.94 ± 19.73	71 (67.6%)	78.84 ± 49.04 days	–	GCS (pre), GOS (post)	25.96 ± 15.61 months
Liang et al. (2007)	Retrospective	TBI (100.0%)	Unilateral (86.9%) Bilateral (13.1%)	23	28.6 (16–41)	18 (78.3%)	5–8 weeks	–	GCS (pre), GOS (post)	1 month
Su et al. (2017)	Retrospective	TBI (100.0%)	Bilateral (31.2%) Unilateral R (37.5%) Unilateral L (31.3%)	16	42.4 ± 15.8	12 (75.0%)	NA	BI	MMSE	31.2 ± 7.5 days

Note

MMSE: Mini‐Mental State Examination; K‐MMSE: Korean Mini‐Mental State Examination; RAVLT: Rey Auditory Verbal Learning Test immediate; RAVLT‐DR: and 30‐min delayed recall; DST: Digit Span Test; GCS: Glasgow Coma Scale; GOS: Glasgow Outcome Scale; CRS‐R: Coma Recovery Scale‐Revised; LCF: Level of Cognitive Functioning Scale; FIM: Functional Independence Measure; K‐BI: Korean Barthel Index; TBI: traumatic brain injury; NA: not available; pre: measured only at baseline; post: measure only at follow‐up.

Four of selected articles included both early and late cranioplasty groups: Two studies (Kim, Kim, & Hyun, 2017; Songara et al., 2016) used 90 days as threshold for dividing patients into early and late groups, whereas two studies (Corallo et al., 2017; Di Stefano et al., 2016) used a threshold of 180 days. For two studies (Honeybul et al., 2016; Jasey et al., 2018), we set at 3 months the threshold to split the patients in two groups, as the median timings were 99 days and 105 days, respectively. Notably, the study population by Jasey et al. (2018) consisted in 26 subjects with a decompressive craniectomy, but only 13 underwent also cranioplasty, who were the only participants included in our analysis.

Half of selected studies were prospective (Di Stefano et al., 2016; Kim et al., 2017; Songara et al., 2016). For 65.44% of patients, the cause of DC was trauma, followed by a cerebrovascular disease (30.86%) and other causes (3.70%). One study included only traumatic brain injury (TBI) patients (Songara et al., 2016). The percentage of trauma was rather homogeneous between early and late patients, 46.40% and 53.60%, respectively.

Cranial procedures locations, when specified, included unilateral, bilateral and bifrontal.

The mean ± *SD* time interval from DC to CP was 146.76 ± 108.46 days, and it was significantly longer in late (195.79 ± 122.62 days) than in early patients (86.08 ± 33.65 days).

The mean age of participants at baseline was 44.62 ± 15.96 years, with no statistically different between patients submitted to early (46.30 ± 16.42 years) or late (43.22 ± 15.51 years) CP.

Only two studies reported complications after cranioplasty (Corallo et al., 2017; Songara et al., 2016), and in both cases, they were observed in patients belonging to the late group.

For the primary outcome, the studies used different assessment tools to evaluate the functional recovery: FIM was used in three studies, MMSE in three studies, DST in three studies, RAVLT and RAVLT‐DR in two studies, and GCS in two studies, whereas LCF, GOS, and CRS‐R in only one study. The interval between assessments after cranioplasty, as well as the number of evaluations, was different among studies: One study performed one follow‐up within 3 days (Honeybul et al., 2016), one study a follow‐up within 4 weeks (Kim et al., 2017), and four studies performed two follow‐up (Corallo et al., 2017; Di Stefano et al., 2016; Jasey et al., 2018; Songara et al., 2016). However, except for one study (Jasey et al., 2018), all authors reported one follow‐up after 1 month from cranioplasty, which was the postcranioplasty evaluation considered in our analysis.

### 3.3 Timing effects in cognitive domain

**Figure 2:**
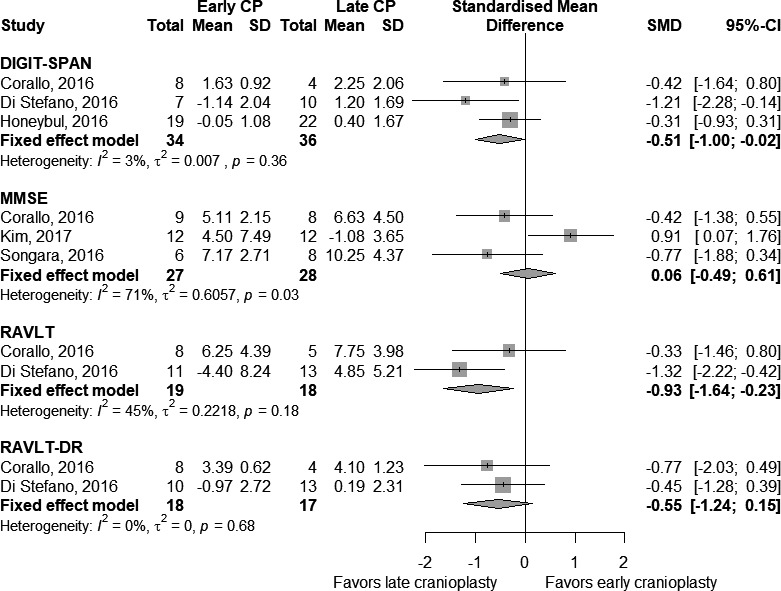
Comparison of early cranioplasty (early CP) versus late cranioplasty (late CP) on pre‐ and postcognitive scores. Number of participants, with mean and standard deviation of changes in MMSE score, is presented for each study in any group. The point estimate and the overall effect, with 95% confidence intervals, are indicated by a diamond in the forest plots

Given that there was no significant heterogeneity for any analyses, a fixed‐effects analysis was used. Figure 2 shows meta‐analyses of early CP versus late CP, subdivided by type of cognitive outcome. There were 55 participants undergoing MMSE in three studies (27 early, 28 late). Here, the estimates of heterogeneity (*τ* ^2^ = 0.61 and *I* ^2^ = 71.3% [2.4%; 91.5%]) indicated a moderate statistical heterogeneity probably due to a bias, as hinted by the corresponding funnel plot. The fixed‐effects model showed that an early procedure did not significantly improve MMSE score (SMD = 0.06 [−0.49; 0.61], *p*‐value = 0.83). Concerning the remaining cognitive tests, instead, 70 participants underwent the DS test in three studies (34 early, 36 late) with a very low statistical heterogeneity (*τ* ^2^ = 0.007 and *I* ^2^ = 3%); 37 participants underwent the RAVLT in two studies (19 early, 18 late) with a moderate statistical heterogeneity (*τ* ^2^ = 0.22 and *I* ^2^ = 45%); and 35 participants underwent the RAVLT‐DR in two studies (18 early, 17 late) with an absent statistical heterogeneity (*τ* ^2^ = 0 and *I* ^2^ = 0%). The corresponding fixed‐effects models showed that a late procedure was more effective in improving memory functions (SMD = −0.63 [−0.97; −0.28], *p*‐value < 0.001).

**Figure 3:**
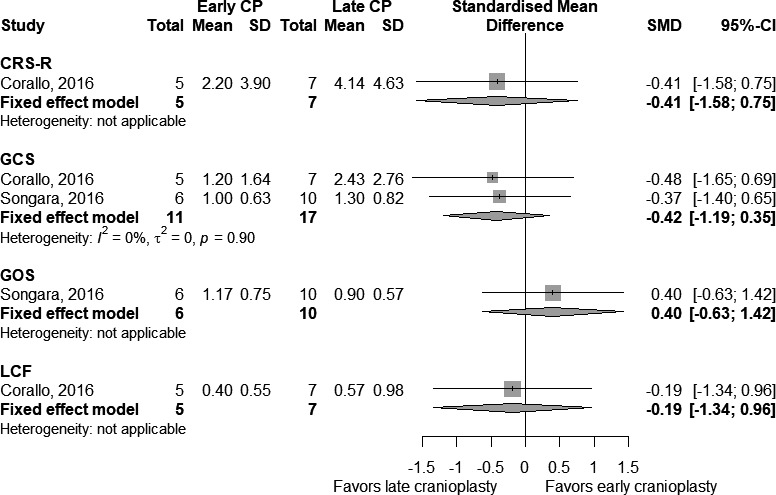
Comparison of early cranioplasty (early CP) versus late cranioplasty (late CP) on pre‐ and postcognitive test scores for postcoma patients. Number of participants, with mean and standard deviation of changes in test score, is presented for each study in any group. The point estimate and the overall effect, with 95% confidence intervals, are indicated by a diamond in the forest plots

Figure 3 shows meta‐analyses of early versus late CP, subdivided by type of cognitive outcome used in postcoma patients. There were 28 participants undergoing GCS in two studies (11 early, 17 late) with an absent statistical heterogeneity (*τ* ^2^ = 0 and *I* ^2^ = 0%). We found that a late procedure was significantly effective in improving the clinical condition compared to an early procedure, although it did not reach the statistical significance (SMD = −0.42 [−1.19; 0.35], *p*‐value = 0.29). All the remaining scales were used in only one study; therefore, no consistent results emerged.

Overall, these results show very strong evidence of the positive effects of cranioplasty on cognitive functions, but independently from the timing (SMD = −0.19 [−0.68; 0.31], *p*‐value = 0.50).

### 3.4 Subgroup analysis between 3 and 6 months

**Figure 4:**
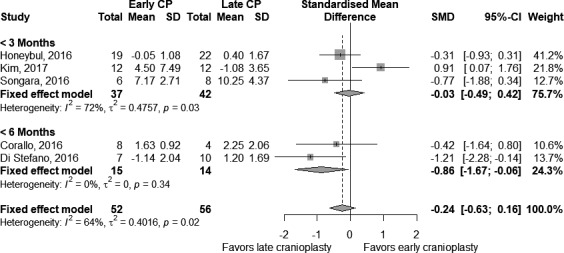
Comparison of early cranioplasty (early CP) versus late cranioplasty (late CP) on pre‐ and postcognitive test scores according to the timing from decompressive craniectomy. Number of participants, with mean and standard deviation of changes in test score, is presented for each study in any group. The point estimate and the overall effect, with 95% confidence intervals, are indicated by a diamond in the forest plots

We subdivided the studies according to the time interval from DC to CP. Three studies set at 3 months the threshold between early and late CP, including 79 participants (37 in the early and 42 in the late group). Results of the meta‐analysis (Figure 4) showed a moderate heterogeneity across studies (*τ* ^2^ = 0.48 and *I* ^2^ = 72%). The fixed‐effects model indicated a nonsignificant difference between early and late CP groups (SMD = −0.03 [−0.49; 0.42], *p*‐value = 0.89). On the contrary, two studies set at 6 months the threshold between early and late CP, including 29 participants (15 in early and 14 in late CP). Here, we found that a late procedure was more effective in improving memory functions (SMD = −0.86 [−1.67; −0.06], *p*‐value = 0.03). However, no statistical significance emerged in the overall model (SMD = −0.24 [−0.63; 0.16], *p*‐value = 0.25).

### 3.5 Timing effects in motor domain

**Figure 5:**
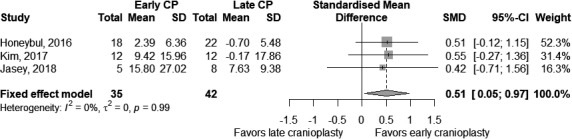
Comparison of early cranioplasty (early CP) versus late cranioplasty (late CP) on pre‐ and postmotor test scores. Number of participants, with mean and standard deviation of changes in test score, is presented for each study in any group. The point estimate and the overall effect, with 95% confidence intervals, are indicated by a diamond in the forest plots

Three studies assess the motor recovery by means of the FIM scale for 77 participants (35 early, 42 late) with an absent statistical heterogeneity (*τ* ^2^ = 0 and *I* ^2^ = 0%). We found that an early procedure was significantly effective in improving the motor functions compared to a late procedure (SMD = 0.51 [0.05; 0.97], *p*‐value = 0.03), as showed in Figure 5.

As all studies set at 3 months the threshold between early and late CP, we did not perform the subgroup analysis.

## 4 DISCUSSION

The optimal cranioplasty timing is a controversial matter. This choice mainly depends on the presence of complications, as well as the time needed for the recovery.

Several studies define “early cranioplasty” as a cranioplasty performed within 91 days from decompressive craniectomy (Malcolm et al., 2018, 2016; Xu et al., 2015). Notably, Xu et al. (2015) sustain that early cranioplasty may reduce the duration of surgery by reducing difficulties in dissecting the scalp flap and fitting the bone flap. Nonetheless, this early procedure cannot reduce the complications and may even increase the risk of hydrocephalus. Indeed, Tasiou et al. reported that delayed cranioplasty should be preferred to minimize the risk of infection that may be caused by intervening in a still contaminated wound (Tasiou et al., 2014). Malcolm et al. (2016) showed that early cranioplasty, with almost certain hydrocephalus management, has similar complication rates to late cranioplasty.

In the last few years, researcher interest is moving toward the association of cranioplasty with the recovery of consciousness and cognitive function as well as the timing of performing cranioplasty (Huang et al., 2013; Shahid et al., 2018; Songara et al., 2016). Rish et al. (1979) reported that cranioplasty performed within 6 months after DC is associated with poor outcomes, Huang et al. (2013) sustained that the timing of cranioplasty is not related to the neurological outcomes of TBI, and Corallo et al. found that the neurological recovery is independent from timing and patient's clinical status. (Shamay‐Tsoory, Tomer, Goldsher, Berger, & Aharon‐Peretz, 2004) However, Malcom et al., in a more recent meta‐analysis, including three motor outcomes and a tool specific for postcoma patients, confirmed the positive effect of cranioplasty on neurological function and claimed that an early procedure may enhance this effect (Malcolm et al., 2018). Similarly, many recent studies recommend early cranioplasty because of its association with clinical improvement (Bender et al., 2013; Chibbaro et al., 2011; Liang et al., 2007; Quah et al., 2016), which can be performed as early as 2 weeks postcraniectomy (and in any case not later than 6 months) to lower the overall cost of care by eliminating the need for additional hospital admissions (Beauchamp et al., 2010). Indeed, it would seem that the majority of neurocognitive changes tend to be at their maximum initially and then decline gradually (Di Stefano et al., 2016), given that ipsilateral low cerebral blood flows increased and reached normal levels after CP (Erdogan et al., 2003), raising the recovery of motor and cognitive functioning (Su et al., 2017).

These contradictory results may be attributed to several factors. First of all the heterogeneity of the population studied, but also the study design features, the choice of surgical approach and operational factors (Sancisi et al., 2009). Thus, our review was aimed at shedding some light on the ongoing debate concerning the right timing to perform cranioplasty and to observe positive effects on cognitive and motor functions. The main question was whether it is reasonable to suggest performing cranioplasty within 90 days from craniectomy to improve the neurological recovery.

Our results showed that such timing is “optimal” only when considering motor outcomes. Indeed, in all studies included in this work, we observed greater positive effects on motor function in the early than late cranioplasty group. On the contrary, to observe a significant cognitive recovery CP should be performed later, although Kim et al. (2017) reported a strong evidence of effects on cognitive functions within 90 days. However, its retrospective study design may lead to a minor reliability since data collected and the measured outcomes are not planned before the study began. Indeed, the follow‐up assessment was performed not “after” but “within” 4 weeks; hence, the recovery could be not evident in all patients, especially by using the MMSE test. Although it is one of the most popular tests in clinical and research settings, this tool is not sensitive enough to detect cognitive recovery, as it is rather a screening test. In our opinion, patients undergoing CP should be evaluated by means of a detailed neurocognitive battery, without lingering on their global recovery often assessed through short evaluations. Indeed, it is necessary to standardize common guideline on what kind of tests should be administered to patients following CP, since the assessment is not homogenous, often because of the different patient's etiology and clinical conditions. In this study, about 65% of patient's disease was trauma, whereas about 30% was cerebrovascular disease. Although the difference in rehabilitation approaches between vascular versus TBI is little (Shamay‐Tsoory et al., 2004), the pathology may affect the timing of performing CP to manage the risk of complications. Even if this issue is not so important in motor functions, it is fundamental in the cognitive domain. To this aim, the assessment should be specifically based on the site and side of lesion, as some brain areas are more strictly related to specific cognitive functions than others (Redolfi et al., 2017).

With regard to the memory tests, the findings suggest that late CP leads to better overall effects. Notably, when focusing on the Digit Span test results, two studies (Corallo et al., 2017; Di Stefano et al., 2016) showed a more significant recovery after 6 months from CP, whereas one study (Honeybul et al., 2016), which has the highest weight, after only 3 months. Thus, we could suppose that a CP performed between 3 and 6 months leads to more significant cognitive recovery, maybe by the restoration of physiological cerebrospinal fluid circulation that, in turn, allows an efficient restoration of blood circulation and, consequently, of the large‐scale neuronal networks responsible for cognition (Corallo et al., 2017; Rish et al., 1979). Indeed, before CP, most of the cognitive abnormalities may be due to changes in cerebrovascular and cerebrospinal fluid hydrodynamics, as per the “sinking skin flap syndrome.” (Coelho et al., 2014; Erdogan et al., 2003; Juul et al., 2000; Maekawa et al., 1999; Mah & Kass, 2016; Winkler et al., 2000). However, it is possible that the early group of Honeybul (Liang et al., 2007) had an improved outcome after CP in a shorter time due to less severely injured patients than those reported in Corallo et al. (2017) and Di Stefano et al. (2016). Moreover, we have to underline that in Honeybul et al. (2016) the follow‐up assessment was performed within 3 days from CP, as opposed to Corallo et al. (2017) and Di Stefano et al. (2016) who performed it after 1 month, thus explaining the substantial difference that might influence the test scores. Indeed, the difference in follow‐up assessment times after CP is another important issue to discuss, since it can affect the measurement. After all, in many studies the greatest improvements were evident many months after cranioplasty and most of the clinical improvement due to cranioplasty is secondary to prolonged effects on brain physiology, rather than immediate changes (Jasey et al., 2018). However, neurorehabilitation programs (if performed) might affect outcomes after longer times (Jolliffe, Lannin, Cadilhac, & Hoffmann, 2018), reinforcing cranioplasty effects on spontaneous cognitive recovery. Su et al. (2017) observed synergetic effects of cranioplasty on TBI patients with rehabilitation training, both in the motor and in the cognitive domains. Moreover, it is well known that an early neuropsychological rehabilitation that has been performed for an adequate time can affect the outcomes in both severe brain injured and patients with disorder of consciousness (Sancisi et al., 2009).

It is noteworthy to highlight that in postcoma patients, results showed very strong evidence of effects of cranioplasty on cognitive functions, but independently from the timing. Unfortunately, our meta‐analysis included only two studies; thus, the findings might not correctly reflect reality. After all, the current literature is poor of studies investigating cranioplasty effects on cognitive functions by means of specific neuropsychological assessment, and, to the best of our knowledge, this is the first attempt to do such analysis.

To summarize, cranioplasty performed within 30 days after initial craniectomy may minimize infection, seizure, and bone flap resorption, whereas waiting >90 days may minimize hydrocephalus but may increase the risk of seizure (Morton et al., 2018; Thavarajah, Lacy, Hussien, & Sugar, 2012). Moreover, at 6‐month follow‐up patients with severe brain injury got better functional outcomes after early than late CP (Yang, Song, Yoon, & Seo, 2018).

A limitation of the study consists in the fact that we did not include the key word “complications” in our database search, although it has been reported that postsurgical complications after cranioplasty may influence the motor and cognitive recovery and the outcome. Thus, further research is needed to address this important issue.

## 5 CONCLUSIONS

Despite the limitations of this meta‐analysis, findings confirm that cranioplasty may improve cognitive and motor recovery. Although 6 months is considered the minimum time to reduce complications, cranioplasty performed within 3 months from decompressive craniectomy may lead to greater effects on motor functions, while for the cognitive domain that the best choice seems to be from three to 6 months, especially if the patient underwent neuropsychological rehabilitation. Future prospective larger sample studies are needed to standardize the best timing of performing CP in patients with different disorders, also by using specific psychometric approaches in order to improve functional recovery and thus patient's quality of life.

## CONFLICT OF INTEREST

The authors declare that they have no financial or other conflict of interests in relation to this research and its publication.

## References

[brb31106-bib-0001] Alibhai, M. K. , Balasundaram, I. , Bridle, C. , & Holmes, S. B. (2013). Is there a therapeutic role for cranioplasty? International Journal of Oral and Maxillofacial Surgery, 42(5), 559–561. 10.1016/j.ijom.2013.01.001 23415243

[brb31106-bib-0002] Aydin, S. , Kucukyuruk, B. , Abuzayed, B. , Aydin, S. , & Sanus, G. Z. (2011). Cranioplasty: Review of materials and techniques. Journal of Neurosciences in Rural Practice., 2(2), 162–167. 10.4103/0976-3147.83584 21897681 PMC3159354

[brb31106-bib-0003] Beauchamp, K. M. , Kashuk, J. , Moore, E. E. , Bolles, G. , Rabb, C. , Seinfeld, J. , … Sauaia, A. (2010). Cranioplasty after postinjury decompressive craniectomy: Is timing of the essence? Journal of Trauma, 69(2), 270–274. 10.1097/TA.0b013e3181e491c2 20699735

[brb31106-bib-0004] Bender, A. , Heulin, S. , Röhrer, S. , Mehrkens, J. H. , Heidecke, V. , Straube, A. , & Pfefferkorn, T. (2013). Early cranioplasty may improve outcome in neurological patients with decompressive craniectomy. Brain Injury, 27(9), 1073–1079. 10.3109/02699052.2013.794972 23662672

[brb31106-bib-0005] Bijlenga, P. , Zumofen, D. , Yilmaz, H. , & Creisson, E. (2007). Orthostatic mesodiencephalic dysfunction after decompressive craniectomy. Journal of Neurology, Neurosurgery & Psychiatry, 78(4), 430–433. 10.1136/jnnp.2006.099242 PMC2077792 17119005

[brb31106-bib-0006] Castaño‐Leon, A. M. , Cepeda, S. , Paredes, I. , Gómez, P. A. , Jiménez‐Roldán, L. , Lagares, A. , & Pérez‐Núñez, A. (2017). Symptomatic ptosis cerebelli after suboccipital craniectomy in a patient with severe brain trauma. Brain Injury, 31(10), 1294–1297. 10.1080/02699052.2017.1309571 28585887

[brb31106-bib-0007] Chibbaro, S. , Di Rocco, F. , Mirone, G. , Fricia, M. , Makiese, O. , Di Emidio, P. , … Bresson, D. (2011). Decompressive craniectomy and early cranioplasty for the management of severe head injury: A prospective multicenter study on 147 patients. World Neurosurgery, 75(3–4), 558–562. 10.1016/j.wneu.2010.10.020 21600512

[brb31106-bib-0008] Cho, Y. J. , & Kang, S. H. (2017). Review of cranioplasty after decompressive craniectomy. Korean Journal of Neurotrauma, 13(1), 9–14. 10.13004/kjnt.2017.13.1.9 28512612 PMC5432454

[brb31106-bib-0009] Coelho, F. , Oliveira, A. M. , Paiva, W. S. , Freire, F. R. , Calado, V. T. , Amorim, R. L. , … Teixeira, M. J. (2014). Comprehensive cognitive and cerebral hemodynamic evaluation after cranioplasty. Neuropsychiatric Disease and Treatment, 10, 695–701. 24833902 10.2147/NDT.S52875 PMC4014378

[brb31106-bib-0010] Collin, C. , Wade, D. T. , Davies, S. , & Horne, V. (1988). The barthel ADL index: A reliability study. International Disability Studies, 10(2), 61–63. 10.3109/09638288809164103 3403500

[brb31106-bib-0011] Corallo, F. , Calabro, R. S. , Leo, A. , & Bramanti, P. (2015). Can cranioplasty be effective in improving cognitive and motor function in patients with chronic disorders of consciousness? A case report. Turkish Neurosurgery, 25(1), 193–196. 25640571 10.5137/1019-5149.JTN.10618-14.2

[brb31106-bib-0012] Corallo, F. , De Cola, M. C. , Lo Buono, V. , Marra, A. , De Luca, R. , Trinchera, A. , … Calabrò, R. S. (2017). Early vs late cranioplasty: What is better? International Journal of Neuroscience., 127(8), 688–693. 10.1080/00207454.2016.1235045 27609482

[brb31106-bib-0013] Corallo, F. , Marra, A. , Bramanti, P. , & Calabrò, R. S. (2014). Effect of cranioplasty on functional and neuro‐psychological recovery after severe acquired brain injury: Fact or fake? Consideration on a single case. Functional Neurology, 29(4), 273–275. 25764258 PMC4370441

[brb31106-bib-0014] Di Stefano, C. , Rinaldesi, M. L. , Quinquinio, C. , Ridolfi, C. , Vallasciani, M. , Sturiale, C. , & Piperno, R. (2016). Neuropsychological changes and cranioplasty: A group analysis. Brain Injury, 30(2), 164–171. 10.3109/02699052.2015.1090013 26647093

[brb31106-bib-0015] Doyle, D. J. (1989). Glasgow coma scale (GCS) In Computer programs in clinical and laboratory medicine (pp. 113–116). New York, NY: Springer.

[brb31106-bib-0016] Erdogan, E. , Düz, B. , Kocaoglu, M. , Izci, Y. , Sirin, S. , & Timurkaynak, E. (2003). The effect of cranioplasty on cerebral hemodynamics: Evaluation with transcranial Doppler sonography. Neurology India, 51(4), 479–481. 14742926

[brb31106-bib-0017] Folstein, M. F. , Folstein, S. E. , & McHugh, P. R. (1975). ‘Mini‐mental state’: A practical method for grading the cognitive state of patients for the clinician. Journal of Psychiatric Research, 12, 189–198. 10.1016/0022-3956(75)90026-6 1202204

[brb31106-bib-0018] Giacino, J. T. , Kalmar, K. , & Whyte, J. (2004). The JFK coma recovery scale‐revised: Measurement characteristics and diagnostic utility. Archives of Physical Medicine and Rehabilitation, 85(12), 2020–2029. 15605342 10.1016/j.apmr.2004.02.033

[brb31106-bib-0019] Hofmeijer, J. , Kappelle, L. J. , Algra, A. , Amelink, G. J. , van Gijn, J. , & van der Worp, H. B. (2009). Surgical decompression for space‐occupying cerebral infarction (the Hemicraniectomy After Middle Cerebral Artery infarction with Life‐threatening Edema Trial [HAMLET]): A multicentre, open, randomised trial. Lancet Neurology, 8(4), 326–333. 10.1016/S1474-4422(09)70047-X 19269254

[brb31106-bib-0020] Honeybul, S. , & Ho, K. M. (2016). Cranioplasty: Morbidity and failure. British Journal of Neurosurgery, 30(5), 523–528. 10.1080/02688697.2016.1187259 27215939

[brb31106-bib-0021] Honeybul, S. , Janzen, C. , Kruger, K. , & Ho, K. M. (2013). The impact of cranioplasty on neurological function. British Journal of Neurosurgery, 27(5), 636–641. 10.3109/02688697.2013.817532 23883370

[brb31106-bib-0022] Honeybul, S. , Janzen, C. , Kruger, K. , & Ho, K. M. (2016). The incidence of neurologic susceptibility to a skull defect. World Neurosurgery, 86, 147–152. 10.1016/j.wneu.2015.09.081 26433098

[brb31106-bib-0023] Huang, Y. H. , Lee, T. C. , Yang, K. Y. , & Liao, C. C. (2013). Is timing of cranioplasty following posttraumatic craniectomy related to neurological outcome? International Journal of Surgery, 11(9), 886–890. 10.1016/j.ijsu.2013.07.013 23933129

[brb31106-bib-0024] Jasey, N. , Ward, I. , Lequerica, A. , & Chiaravalloti, N. D. (2018). The therapeutic value of cranioplasty in individuals with brain injury. Brain Injury, 32(3), 318–324. 10.1080/02699052.2017.1419283 29283285

[brb31106-bib-0025] Jeyaraj, P. (2015). Importance of early cranioplasty in reversing the syndrome of the trephine/motor trephine syndrome/sinking skin flap syndrome. Journal of Maxillofacial and Oral Surgery, 14(3), 666–673. 10.1007/s12663-014-0673-1 26225060 PMC4510081

[brb31106-bib-0026] Jolliffe, L. , Lannin, N. A. , Cadilhac, D. A. , & Hoffmann, T. (2018). Systematic review of clinical practice guidelines to identify recommendations for rehabilitation after stroke and other acquired brain injuries. British Medical Journal Open, 8(2), e018791 10.1136/bmjopen-2017-018791 PMC5855444 29490958

[brb31106-bib-0027] Juul, N. , Morris, G. F. , Marshall, S. B. , & Marshall, L. F. (2000). Intracranial hypertension and cerebral perfusion pressure: Influence on neurological deterioration and outcome in severe head injury. Journal of Neurosurgery, 92(1), 1–6. 10.3171/jns.2000.92.1.0001 10616075

[brb31106-bib-0028] Keith, R. A. (1987). The functional independence measure: A new tool for rehabilitation. Advances in Clinical Rehabilitation, 1, 6–18. 3503663

[brb31106-bib-0029] Kim, B. W. , Kim, T. U. , & Hyun, J. K. (2017). Effects of early cranioplasty on the restoration of cognitive and functional impairments. Annals of Rehabilitation Medicine, 41(3), 354–361. 10.5535/arm.2017.41.3.354 28758072 PMC5532340

[brb31106-bib-0030] Landis, J. R. , & Koch, G. G. (1977). The measurement of observed agreement for categorical data. Biometrics, 33, 159–174. 843571

[brb31106-bib-0031] Liang, W. , Xiaofeng, Y. , Weiguo, L. , Gang, S. , Xuesheng, Z. , Fei, C. , & Gu, L. (2007). Cranioplasty of large cranial defect at an early stage after decompressive craniectomy performed for severe head trauma. Journal of Craniofacial Surgery, 18(3), 526–532. 10.1097/scs.0b013e3180534348 17538313

[brb31106-bib-0032] Maekawa, M. , Awaya, S. , & Teramoto, A. (1999). Cerebral blood flow before and after cranioplasty performed during the chronic stage after decompressive craniectomy evaluated by Xenon‐enhanced computerized tomography CBF scanning. No Shinkei Geka, 27, 717–722. 10457935

[brb31106-bib-0033] Mah, J. K. , & Kass, R. A. (2016). The impact of cranioplasty on cerebral blood flow and its correlation with clinical outcome in patients underwent decompressive craniectomy. Asian Journal of Neurosurgery, 11(1), 15–21. 26889273 10.4103/1793-5482.172593 PMC4732236

[brb31106-bib-0034] Malcolm, J. G. , Rindler, R. S. , Chu, J. K. , Chokshi, F. , Grossberg, J. A. , Pradilla, G. , & Ahmad, F. U. (2018). Early cranioplasty is associated with greater neurological improvement: A systematic review and meta‐analysis. Neurosurgery, 82(3), 278–288. 10.1093/neuros/nyx182 28419358

[brb31106-bib-0035] Malcolm, J. G. , Rindler, R. S. , Chu, J. K. , Grossberg, J. A. , Pradilla, G. , & Ahmad, F. U. (2016). Complications following cranioplasty and relationship to timing: A systematic review and meta‐analysis. Journal of Clinical Neuroscience, 33, 39–51. 10.1016/j.jocn.2016.04.017 27499122

[brb31106-bib-0036] McMinn, M. R. , Wiens, A. N. , & Crossen, J. R. (1988). Rey auditory‐verbal learning test: Development of norms for healthy young adults. Clinical Neuropsychologist, 2(1), 67–87. 10.1080/13854048808520087

[brb31106-bib-0037] Morton, R. P. , Abecassis, I. J. , Hanson, J. F. , Barber, J. K. , Chen, M. , Kelly, C. M. , … Chesnut, R. M. (2018). Timing of cranioplasty: A 10.75‐year single‐center analysis of 754 patients. Journal of Neurosurgery, 128(6), 1648–1652. 10.3171/2016.11.JNS161917 28799868

[brb31106-bib-0038] Nalbach, S. V. , Ropper, A. E. , Dunn, I. F. , & Gormley, W. B. (2012). Craniectomy‐associated Progressive Extra‐Axial Collections with Treated Hydrocephalus (CAPECTH): Redefining a common complication of decompressive craniectomy. Journal of Clinical Neuroscience, 19(9), 1222–1227. 10.1016/j.jocn.2012.01.016 22727206

[brb31106-bib-0039] Nguyen, H. S. , Doan, N. B. , Gelsomino, M. J. , Shabani, S. , & Mueller, W. M. (2016). Good outcomes in a patient with a Duret hemorrhage from an acute subdural hematoma. International Medical Case Reports Journal, 27(9), 15–18. 10.2147/IMCRJ.S95809 PMC4734784 26869816

[brb31106-bib-0040] Quah, B. L. , Low, H. L. , Wilson, M. H. , Bimpis, A. , Nga, V. D. W. , Lwin, S. , … Salek, M. A. A. (2016). Is there an optimal time for performing cranioplasties? Results from a prospective multinational study. World Neurosurgery, 94, 13–17. 10.1016/j.wneu.2016.06.081 27368511

[brb31106-bib-0041] Ratnasingam, D. , Lovick, D. S. , Weber, D. M. , Buonocore, R. V. , & Williams, W. V. (2015). An unusual recovery from traumatic brain injury in a young man. The Linacre Quarterly, 82(1), 55–66. 10.1179/2050854914Y.0000000030 25698843 PMC4313431

[brb31106-bib-0042] Redolfi, A. , Gugliotta, M. , Borsotti, M. , D’Amato, A. , Gemignani, P. , Maietti, A. , … Mazzucchi, A. (2017). Long‐term services for the care and rehabilitation of people with severe acquired brain injury: A multicentre, cross‐sectional study of 536 Italian families. Annali Dell'istituto Superiore Di Sanità, 53(3), 253–265. 10.4415/ANN_17_03_12 28956806

[brb31106-bib-0043] Rish, B. L. , Dillon, J. D. , Meirowsky, A. M. , Caveness, W. F. , Mohr, J. P. , Kistler, J. P. , & Weiss, G. H. (1979). Cranioplasty: A review of 1030 cases of penetrating head injury. Neurosurgery, 4, 381–385. 10.1097/00006123-197905000-00002 111153

[brb31106-bib-0044] Sancisi, E. , Battistini, A. , Di Stefano, C. , Simoncini, L. , Simoncini, L. , Montagna, P. , & Piperno, R. (2009). Late recovery from post‐traumatic vegetative state. Brain Injury, 23(2), 163–166. 10.1080/02699050802660446 19191095

[brb31106-bib-0045] Sander, A. (2012). The level of cognitive functioning scale. The Center for Outcome Measurement in Brain Injury. Retrieved from https://www.tbims.org/combi/lcfs (accessed April 27, 2018).

[brb31106-bib-0046] Sands, M. L. , & Murphy, J. R. (1996). Use of kappa statistic in determining validity of quality filtering for meta‐analysis: A case study of the health effects of electromagnetic radiation. Journal of Clinical Epidemiology, 49(9), 1045–1051. 10.1016/0895-4356(96)00058-3 8780615

[brb31106-bib-0047] Schroeder, R. W. , Twumasi‐Ankrah, P. , Baade, L. E. , & Marshall, P. S. (2012). Reliable digit span a systematic review and cross‐validation study. Assessment, 9(1), 21–30. 10.1177/1073191111428764 22156721

[brb31106-bib-0048] Segal, D. H. , Oppenheim, J. S. , & Murovic, J. A. (1994). Neurological recovery after cranioplasty. Neurosurgery, 34(4), 729–731. 8008174 10.1227/00006123-199404000-00024

[brb31106-bib-0049] Shahid, A. H. , Mohanty, M. , Singla, N. , Mittal, B. R. , & Gupta, S. K. (2018). The effect of cranioplasty following decompressive craniectomy on cerebral blood perfusion, neurological, and cognitive outcome. Journal of Neurosurgery, 128(1), 229–235. 10.3171/2016.10.JNS16678 28298042

[brb31106-bib-0050] Shamay‐Tsoory, S. G. , Tomer, R. , Goldsher, D. , Berger, B. D. , & Aharon‐Peretz, J. (2004). Impairment in cognitive and affective empathy in patients with brain lesions: Anatomical and cognitive correlates. Journal of Clinical and Experimental Neuropsychology, 26(8), 1113–1127. 10.1080/13803390490515531 15590464

[brb31106-bib-0051] Songara, A. , Gupta, R. , Jain, N. , Rege, S. , & Masand, R. (2016). Early cranioplasty in patients with posttraumatic decompressive craniectomy and its correlation with changes in cerebral perfusion parameters and neurocognitive outcome. World Neurosurgery, 94, 303–308. 10.1016/j.wneu.2016.07.003 27418533

[brb31106-bib-0052] Stelling, H. , Graham, L. , & Mitchell, P. (2011). Does cranioplasty following decompressive craniectomy improve consciousness? British Journal of Neurosurgery, 25(3), 407–409. 10.3109/02688697.2011.566385 21501062

[brb31106-bib-0054] Stiver, S. I. , Wintermark, M. , & Manley, G. T. (2008). Motor trephine syndrome: A mechanistic hypothesis. Acta Neurochirurgica Supplements, 102, 273–277. 19388328 10.1007/978-3-211-85578-2_51

[brb31106-bib-0055] Stiver, S. I. , Wintermark, M. , & Manley, G. T. (2008). Reversible monoparesis following decompressive hemicraniectomy for traumatic brain injury. Journal of Neurosurgery, 109(2), 245–254. 10.3171/JNS/2008/109/8/0245 18671636

[brb31106-bib-0056] Su, J. H. , Wu, Y. H. , Guo, N. W. , Huang, C. F. , Li, C. F. , Chen, C. H. , & Huang, M. H. (2017). The effect of cranioplasty in cognitive and functional improvement: Experience of post‐traumatic brain injury inpatient rehabilitation. Kaohsiung Journal of Medical Sciences, 33(7), 344–350. 10.1016/j.kjms.2017.05.002 28738975 PMC11916026

[brb31106-bib-0057] Tasiou, A. , Vagkopoulos, K. , Georgiadis, I. , Brotis, A. G. , Gatos, H. , & Fountas, K. N. (2014). Cranioplasty optimal timing in cases of decompressive craniectomy after severe head injury: A systematic literature review. Interdisciplinary Neurosurgery, 1, 107–111. 10.1016/j.inat.2014.06.005

[brb31106-bib-0058] Thavarajah, D. , De Lacy, P. , Hussien, A. , & Sugar, A. (2012). The minimum time for cranioplasty insertion from craniectomy is six months to reduce risk of infection–a case series of 82 patients. British Journal of Neurosurgery, 26(1), 78–80. 10.3109/02688697.2011.603850 21973063

[brb31106-bib-0059] Vahedi, K. , Hofmeijer, J. , Juettler, E. , Vicaut, E. , George, B. , Algra, A. , … Hacke, W. (2007). Early decompressive surgery in malignant infarction of the middle cerebral artery: A pooled analysis of three randomised controlled trials. Lancet Neurology, 6(3), 215–222. 10.1016/S1474-4422(07)70036-4 17303527

[brb31106-bib-0060] Wilson, J. T. L. , Pettigrew, L. E. L. , & Teasdale, G. M. (1998). Structured interviews for the Glasgow Outcome Scale and the extended Glasgow Outcome Scale: Guidelines for their use. Journal of Neurotrauma, 15(8), 573–585. 10.1089/neu.1998.15.573 9726257

[brb31106-bib-0061] Winkler, P. A. , Stummer, W. , Linke, R. , Krishnan, K. G. , & Tatsch, K. (2000). The influence of cranioplasty on postural blood flow regulation, cerebrovascular reserve capacity, and cerebral glucose metabolism. Journal of Neurosurgery, 93(1), 53–61. 10.3171/jns.2000.93.1.0053 10883905

[brb31106-bib-0062] Xu, H. , Niu, C. , Fu, X. , Ding, W. , Ling, S. , Jiang, X. , & Early, J. Y. (2015). cranioplasty vs. late cranioplasty for the treatment of cranial defect: A systematic review. Clinical Neurology and Neurosurgery, 136, 33–40. 10.1016/j.clineuro.2015.05.031 26056810

[brb31106-bib-0063] Yang, N. R. , Song, J. , Yoon, K. W. , & Seo, E. K. (2018). How early can we perform cranioplasty for traumatic brain injury after decompressive craniectomy? A retrospective multicenter study. World Neurosurgery, 110, e160–e167. 10.1016/j.wneu.2017.10.117 29101076

[brb31106-bib-0064] Zanaty, M. , Chalouhi, N. , Starke, R. M. , Clark, S. W. , Bovenzi, C. D. , Saigh, M. , … Tjoumakaris, S. I. (2015). Complications following cranioplasty: Incidence and predictors in 348 cases. Journal of Neurosurgery, 123(1), 182–188. 10.3171/2014.9.JNS14405 25768830

[brb31106-bib-0065] Zanotti, B. , Zingaretti, N. , Verlicchi, A. , Robiony, M. , Alfieri, A. , & Parodi, P. C. (2016). Cranioplasty: Review of materials. Journal of Craniofacial Surgery, 27(8), 2061–2072. 10.1097/SCS.0000000000003025 28005754

